# An outbreak of neurologic symptoms among patients exposed to an unknown stench in a high school near an industrial complex: an epidemiological investigation

**DOI:** 10.4178/epih.e2022105

**Published:** 2022-11-09

**Authors:** Kiook Baek, Seongmin Jo, Chulyong Park, Joon Sakong

**Affiliations:** 1 Department of Occupational and Environmental Medicine, Yeungnam University Hospital, Daegu, Korea; 2 Department of Preventive Medicine and Public Health, Yeungnam University Collage of Medicine, Daegu, Korea

**Keywords:** Environmental pollution, Disease outbreaks, Epidemiologic investigation, Students, Neurologic manifestations

## Abstract

**OBJECTIVES:**

Seven students at a high school in Korea visited the emergency room with non-specific neurological symptoms after a stench was noticed during a school entrance ceremony. In relation to this incident, 105 patients visited medical institutions over 5 days. A team of environmental and epidemiological experts was assembled to investigate the incident.

**METHODS:**

Our team of experts participated in the investigation 1 month post-incident. Previously, only air samples had been analyzed. We received results of air samples analyzed by other investigators, medical records of some students, and data from police interviews of patients. Additional investigation and interviews were conducted, and the events were reconstructed in spatial and temporal order.

**RESULTS:**

A cluster of patients was observed on the south side and parts of the north side of the upper floor. A stench like that reported during the incident had been noticed for about 2 years near the school. Students consistently described a similar stench occurring frequently in the vicinity of the school. According to student statements, the odor mainly resembled something burning. The carboxyhemoglobin levels of some students were observed to be >1.5%.

**CONCLUSIONS:**

In the investigation, 2 suspected sources were identified: a science room storing chemicals downstairs from the auditorium and various industrial facilities near the school. Combining the scattered evidence, we considered a toxic puff of gas, perhaps from brief incineration or leakage in a specific area, to be the likely cause of the incident. We describe our approach and the limitations encountered during the investigation.

## GRAPHICAL ABSTRACT

[Fig f5-epih-44-e2022105]

## INTRODUCTION

In 2019, at a high school near an industrial complex in Daegu, Korea, students developed neurological symptoms after a stench of unknown cause was noted during an entrance ceremony. The stench was traced to a specific area of the auditorium. After the event, 7 students visited the emergency room with reports of non-specific neurological symptoms, and 105 students visited medical facilities over 5 days in relation to the incident.

Air pollution and odors from industrial complexes can have various health effects on area residents [1]. Substances such as ammonia, sulfur compounds, and volatile organic compounds (VOCs) can cause odors [2,3]. The acute and chronic health effects of odor exposure can be difficult to ascertain [4], since different pollutants are generated by different factories. Investigators also often experience difficulty predicting the concentration and flow of odors depending on the atmospheric environment [5,6]. Several chemical accidents at industrial facilities have been reported, including a methyl isocyanate leak in Bhopal, India [4] and a styrene leak from a polymer plant in Visakhapatnam, India [7]. In Korea, past chemical-related accidents include a hydrofluoric acid gas leak in Gumi [8,9], a polyethylene plant explosion at the Yeosu National Industrial Complex, and a silicon tetrachloride exposure accident in Gunsan [10]. Many reports of problematic odors have been generated near industrial complexes regardless of accidents [11–13] and without a clear source [14]. However, most accidents with acute health impacts were catastrophic and had an identified source. Here, we present a rare case of more than 100 students who received treatment for suspected poisoning without an identified source.

## MATERIALS AND METHODS

### Overview

The affected high school is located in a hilly area near an industrial complex, at a higher altitude (about 40 m above sea level) than the nearby industrial area (about 25 m above sea level). All students were female. The day of the incident (September 2) was cloudy and wet, with a small amount of rain (3 mm) reported by a meteorological agency in Daegu. The wind direction was northeast. The entrance ceremony was held in the auditorium. During the event, 7 students reported severe headaches and nausea along with noticing a sudden stench in a specific area of the auditorium and consequently visited the emergency room. Other students continued to report symptoms, 74 of whom visited the hospital that day. All other students returned home early. Symptoms continued to develop in the student population, and 105 patients received treatment over 5 days. Initially, the police and atmospheric experts investigated this situation. One month after the incident, an investigation team was convened with 7 members: 2 experts on the air environment; 1 expert each from the chemical, industrial hygiene, sampling and analysis, and medical fields; and 1 non-governmental organization member. One member of our research team joined the investigation team as a medical expert.

### Interviews

Interviews were conducted with patients who experienced symptoms and noticed the stench. The initial interview was conducted by police among the 74 students who visited the hospital on the day of the incident. The questions covered topics such as student location in the auditorium, time of symptom onset, description of the smell, any past experiences of a similar stench, weather conditions, description of symptoms and the name of the hospital visited, length of hospital stay, and health status at the time of the interview. However, since this interview was not conducted by medical or environmental experts, analysis was limited, as the answers were vague and unstructured. The research team conducted additional interviews with some students 1 month later to obtain detailed temporal and spatial descriptions. We gained limited access to the medical records of patients who consented and provided their records. The number of daily clinic or hospital visits and chief concerns were investigated by the school.

### Temporal and spatial analysis

The interview records were analyzed and reconstructed by time series. To spatially place each patient, the locations of the students during, before, and after the event were mapped based on their affiliations within the school. A potential source near the initial cluster was investigated, as was the industrial complex near the school.

### Air sample measurements

Air samples were collected and analyzed by various organizations, and the research team received the results during the joint investigation. Sampling and analysis were conducted based on the possibility of indoor and outdoor pollutants. The location and time of sampling and analysis by each institution are described in Supplementary Material 1.

### Ethics statement

The authors obtained approval from the Yeungnam University Hospital Institutional Review Board (IRB No. 2022-04-040).

## RESULTS

### General characteristics of the exposed group

Approximately 800 students, all female, and school staff were in the auditorium during the ceremony. In Korea, second-year students in high school are 16–17 years old, and third-year students are 17–18 years old. In the present study, the lower and upper floors of the auditorium were on the fourth and fifth floors of the building, respectively. Since the first-year students were on the lower floor and the second-year and third-year students were on the upper floor, one-third of the student population is estimated to have been on the lower floor and two-thirds on the upper floor. The exact number of people in each space at the time was not investigated.

### Patient status

**Figure 1:**
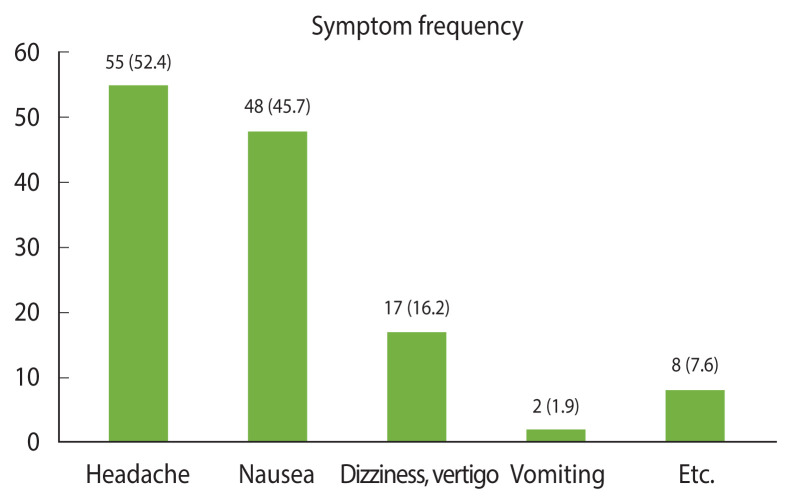
Distribution of symptoms in patients. Multiple symptoms may be present per person. Values are presented as number (%).

The number of medical visits by date is presented in Table 1. Of these patients, 93 (88.6%) were in the second year. The patients reported mainly non-specific neurological symptoms such as headache (52.4%), nausea (45.7%), and dizziness/vertigo (16.2%) (Figure 1). The causative chemical was unknown, and the symptoms were diverse; therefore, diagnosis and treatment varied among the students. None of the students received intensive care or suffered serious sequelae or death, and all recovered. Eight patients underwent carboxyhemoglobin (COHb) analysis and received hyperbaric oxygen therapy. The COHb concentrations of the 8 patients were 1.6±0.2% (range, 1.3 to 2.1), which improved after hyperbaric oxygen treatment.

### Temporal features

From 7:50 to 8:10 on September 2, some students cleaned and opened the windows on the upper floor, and the wind band was practicing on the lower floor of the auditorium. From 8:10 to 9:20, physical education classes were held in the auditorium. At that time, no symptoms or stench were reported. Students entered the auditorium at 9:20 for the event and began to describe a stench, coming primarily from the upper floor area on the south side of the auditorium. The open windows were closed and then opened again, but the strong stench persisted. The stench was also detected on the north side of the auditorium, and after the smell was first noticed, some of the windows on the north side were opened. Most of the teachers who entered after 9:40 and the students on the lower floor did not notice the stench. The event continued from 9:45 to 10:05. Seven students reported symptoms during the event, and symptoms persisted after the event was over. The 7 students visited the emergency room via ambulance. The rest of the students returned to their classrooms, but symptoms continued to develop among the student population. The school conducted a screening, and 74 students were identified as symptomatic. At 14:50, all students were dismissed. Over subsequent days, patients continued to report symptoms and visited outpatient clinics or the hospital. Some students left school early or were absent without visiting the hospital. Since then, several reports have been made of stench in the school, but no symptoms have been noted. The timelines of the day of incident is presented in Table 1, and its aftermath are organized in Supplementary Material 2.

Interviews with teachers about previous situations revealed that an incident involving a similar stench occurred at a nearby school on September 22, 2017. In addition, reports of an intermittent severe stench had been repeatedly raised. The timeline for these incidents is outlined in Table 2.

### Spatial features

**Figure 2:**
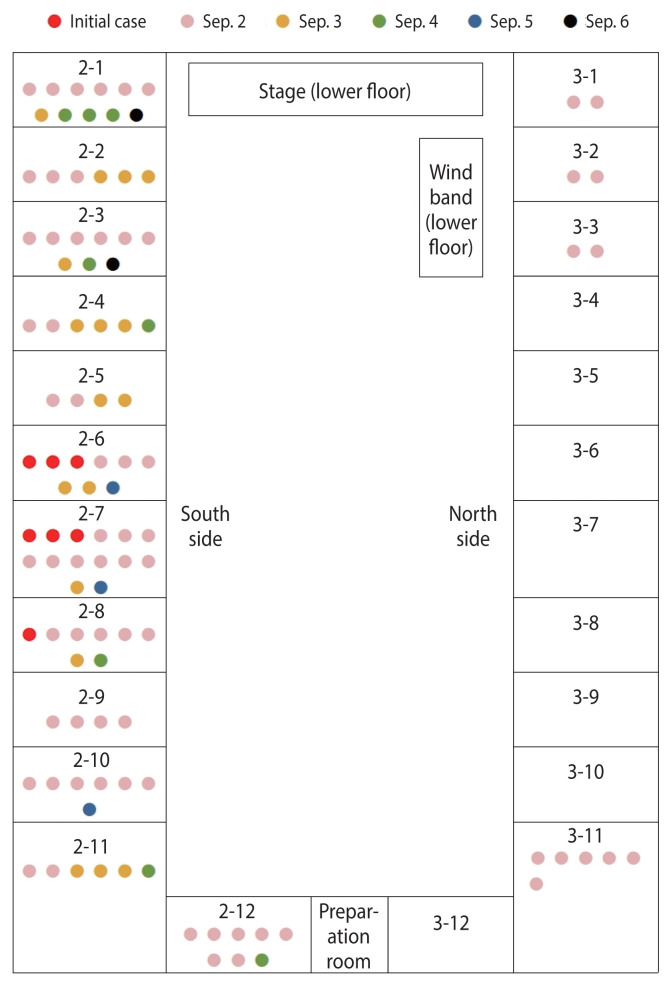
Dot mapping for spatial consideration of patient distribution in the auditorium.

Dot mapping was done among patients who visited the hospital between September 2 and September 6. No patient was identified among the first-year students on the lower floor. Approximately 20 students were in a single class, although this was not indicated (Figure 2). The initial patients were clustered in the middle-south part of the upper floor of the auditorium. On the day of the incident, symptoms occurred mainly among second-year students, but some third-year students on the northwest side also developed symptoms. The symptomatic third-year students were near the location where the stench was noticed on the upper floor.

Since classes and other routine events were held in the classrooms before and after the event in the auditorium, mapping was conducted based on each student’s usual classroom location to spatially describe other cases within the school. The school and auditorium are in separate buildings. The initial patients and those who presented with symptoms on the following day were spatially dispersed relative to the auditorium mapping (Supplementary Material 3).

**Figure 3:**
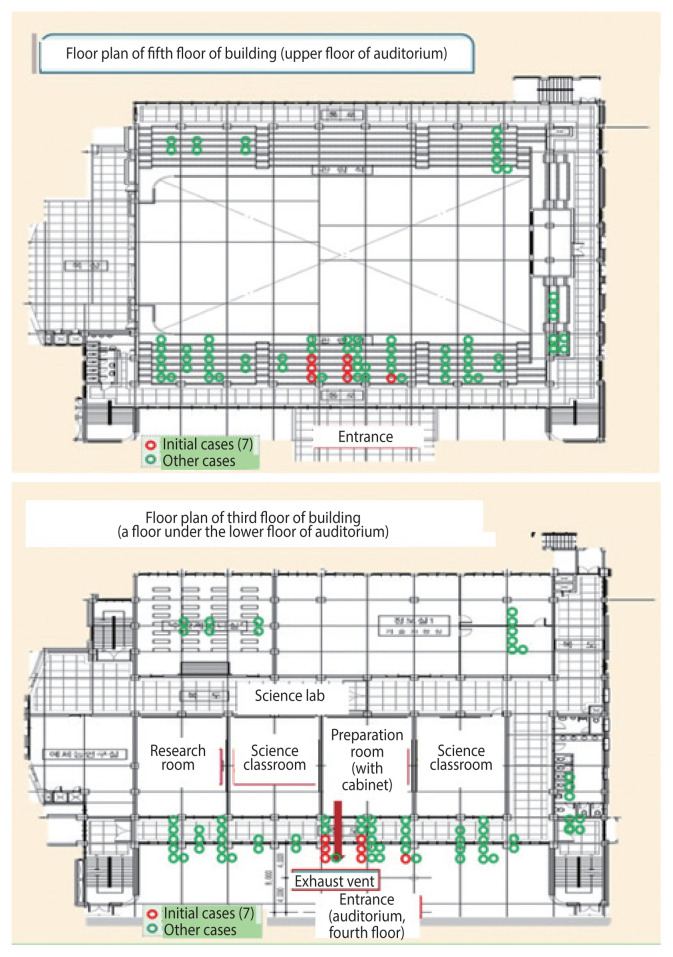
Floor plans of the building’s fifth floor (upper floor of the auditorium, where the initial patient cluster was located) and third floor (under the lower floor of the auditorium).

To investigate and estimate the source of the stench, investigators mapped the school facilities near the auditorium and industrial complexes in the vicinity. Figure 3 shows the floor plan where a science lab is located (the third floor) and the floor where the initial patients were reported (the fifth floor). The science lab was on the lower floor of the auditorium, with the initial cluster of 7 patients located near an exhaust vent from a reagent cabinet (Supplementary Material 4).

**Figure 4:**
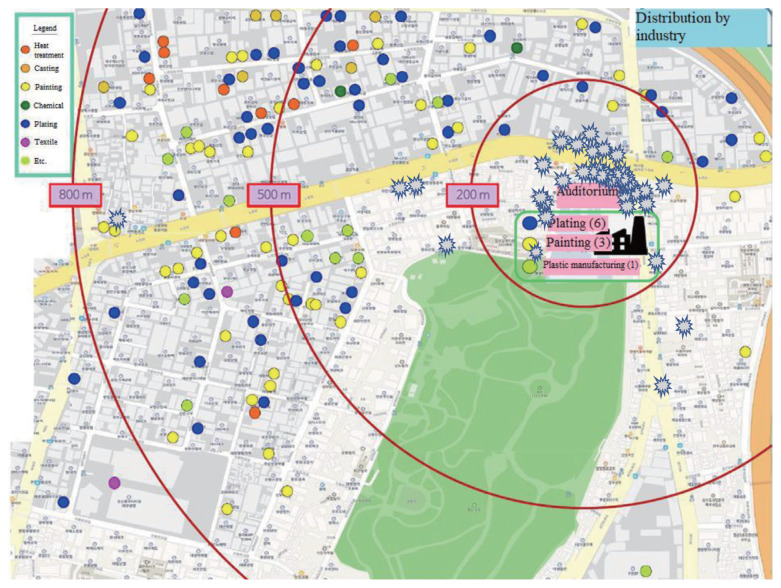
Location of factories near the school by sectors (circle) and places where students had perceived a stench similar to that of the day of the incident (star mark) in the industrial complex near the school.

The school is near an industrial complex that houses various factories, such as plating and casting facilities, that can discharge harmful substances. Second-year students were presented with a map of the school area, then told to mark any location that had a similar stench on the day of the incident (Figure 4). The hypothetical movement of gas in the auditorium that day is represented in Supplementary Material 5. The figure incorporates the wind direction of the day, the location(s) where any similar stench had been sensed before, and the dot mapping of the auditorium.

### Air sampling and analysis

Various measurements were performed at several institutions, and the results are presented in Supplementary Material 2. Information was shared for representative or specific results. No chemicals known to cause serious health effects were detected.

In brief, the result of the Institute of Health and Environment Research shows that the levels of acetaldehyde, acetonitrile, and carbonyl sulfur compounds were partially correlated between the auditorium and the science laboratory. Investigators who performed the sampling claimed that the smell in the science lab resembled that in the auditorium, although smells were not compared on the day of the incident. In the air sampling around the school conducted by the National Institute of Environmental Research, the odorous substances detected in the industrial complex were detected at higher concentrations in the auditorium than in the science lab.

### Other interview findings

Among the 74 patients from the first day of the incident, 8 students were located on the lower floor and 62 students were on the upper floor during the event. Four students gave ambiguous statements about their location “at the time” (police did not specify whether “at the time” referred to the onset of symptoms or recognition of the stench). Although 4 patients who were on the lower floor described themselves as second-year students (and mapping was therefore performed according to that affiliation), they were on the lower floor participating in the wind band. Four of the patients on the lower floor experienced symptoms without recognizing a particular smell, while all patients on the upper floor recognized the stench. The exact mapping was not verified, since the questions and answers were vague. Regarding the stench pattern, 74 people provided subjective statements. The results are shown in Table 3.

The science teacher in charge of the science laboratory had not used the chemicals since the previous May, and no unusual odor was detected in the laboratory. The investigation of the reagent cabinet by the police forensic team yielded no specific findings.

## DISCUSSION

Two sources were suspected as potential causes of the incident by a joint investigation team. The first was toxic puff gas from a nearby industrial complex, and the second was a chemical leak from a cabinet in the science laboratory. We determined that toxic puff gas from an industrial complex was the more likely of the possible causes. The stench was recognized, and symptoms developed in a specific time and space. No one who used that space before 9:20 developed symptoms, and teachers who entered the auditorium after 9:40 were hardly aware of the smell. Most of the patients were second-year students who had been sitting on the south side of the upper floor of the auditorium or third-year students who had been sitting on the north side. Few people on the lower floor reported a stench or symptoms. Accordingly, temporarily generated puff-type toxic gas, rather than plume gas, may have entered through the window on the upper floor and passed through without being scattered to the lower floor.

In the past, students frequently smelled a similar stench in nearby areas. The odor appeared primarily between 17:00 and 19:00, and it seems to have been caused by air pollution due to a temperature inversion. According to interview responses and meteorological data, the day of the incident was cloudy and slightly rainy. A region of low atmospheric pressure had likely formed in the area. Even after the windows on the upper floor of the auditorium were opened for ventilation, the stench persisted. The patients were in the northeast, south, and northwest areas of the auditorium. Based on the meteorological data from the day of the incident, a northeasterly wind seems to have been present in the area. We can reasonably assume that the puff of gas that flowed in from the northeast window of the auditorium traveled northwest through the southern window and affected the students at their respective locations. Based on the combined results of the investigation, we present the hypothetical path of puff gas in the auditorium and the location of the potential emission source in Supplementary Material 5.

After the incident, several substances, such as methyl ethyl ketone (MEK), were detected at high levels in the school and at nearby industrial complexes. This indirectly suggests that the air from the surrounding industrial complex could impact the school. Therefore, the gas may have originated from the industrial complex, potentially (as is our belief) via incineration. Many students testified that the stench smelled like something burning. Among most of the students who underwent COHb analysis, COHb levels were elevated relative to the normal range; additionally, symptoms improved with hyperbaric oxygen chamber therapy [15–17]. According to medical records, the COHb concentration in 7 of 8 patients exceeded 1.5%, which is the upper threshold of the normal range for the average child or adolescent [18]. Carbon monoxide (CO), a representative gas produced by incomplete combustion, may be a component of puff gas generated from material incineration [19]. However, CO is colorless and odorless, and oxygen therapy is an effective treatment for neurological symptoms of various causes [20,21]. As the COHb level in urban areas is higher than in the general population [22], this piece of evidence is not decisive.

An alternative hypothesis, suggested by other members of the investigation team, was that the chemicals originated from the science lab inside the school. The chemical cabinet exhaust vents are located below the area where the initial patient cluster was identified. Analysis of an air sample from the school under weather conditions similar to the day of the incident showed a correlation between substances detected on the upper floor of the auditorium and in the science laboratory, and the smell in the auditorium was similar to that in the science room during sampling. However, few patients were on the lower floor of the auditorium, which is closer to the science room than the upper floor. No damage or leakage of chemicals was identified in the science laboratory. Although the stenches in the science room and the auditorium were similar at the time of sampling, the students stated that a similar stench occurred frequently in the industrial area near the school. Furthermore, no accidents in the science lab, such as leaks or fires, occurred on that day. If a continuous leak had been present in the science lab rather than a single accident, the students who used the auditorium in the morning would have noticed the smell; however, the students who used the auditorium before the event did not notice. Some investigators claimed that the auditorium had not been used for a long time, during which time contaminants had accumulated; however, the interview revealed that the auditorium was used on the morning of the incident. This suggests that an influx of an external puff of gas is more likely than gas coming from the science room. Without the air conditions at the time of the incident, the air sampling data and odor similarity are insufficient to assert the cause of the stench.

Even though the interviewers were not experts and the interview questions were vague, some records did not fit epidemiologically. Most patients were second and third-year students, and most were on the upper floor at the time of the episode. However, some second-year patients were on the lower floor for wind band practice at the time of the incident, and 4 reported developing symptoms even though they could not perceive the stench. Contrarily, no cases of first-year students were noted on the lower floor. In the mapping based on patient affiliation, the patients formed a cluster, but it is difficult to spatially explain the appearance of symptoms in only second-year students among students located on the lower floor. The patients’ symptoms were non-specific and were not objectively confirmed with biomarkers, and patients were retrospectively defined by clinic visitation. After the event, as symptoms occurred sporadically, sensitivity may have been increased through social modulation of pain and symptoms, with the non-specific symptoms worsening in the group members [23,24]. In adolescents, psychosocial stress may cause symptoms such as headaches and dizziness. The incident and group reports of symptoms may act as stressors, and secondary symptoms may appear [25]. Thus, some patients may have been misclassified.

Identifying clear sources of the stench was technically challenging. A transient stench event, or “odor spike,” may last for only a few seconds and be caused by wind in a specific direction from a small point source. Transient odor events are difficult to sample and analyze with conventional solid collection methods [26] and hard to model with classical methods [27]. To identify the source, new sampling methods are required such as air capture and multidisciplinary approaches such as spatial analysis considering wind direction and public participation [28]. In this study, students participated and narrowed down the source area by directly mapping similar smelly areas. However, a limitation of the study is that this process did not lead to further investigation.

The measured concentration of an odorant is often much lower than the reference value associated with acute health effects. Therefore, the related discomfort and health effects are often underestimated [29]. Rather than assessing the health effects of each identified or measured chemical, examiners must identify the health effects of the exposed group [30]. Adolescents are vulnerable to environmental pollutants, and high school students in Korea frequently spend 14 hours or more per day at school [31]. As incidents of continuous exposure to odors have been previously noted in this area, we must continuously identify and update potential health risks through the establishment of a cohort of students and nearby residents [32]. According to the law of the Korea, when a chemical accident occurs, it must be immediately reported and emergency measures must be taken. These include alerting the fire department and the Environment Agency and disclosing the status of the accident situation. Chemical accidents are monitored 24 hours per day. Accident-related regulations are in place to provide an on-site response by the fire department and evacuation guidance for nearby residents by local governments; to predict damage to the accident area; and to provide information on disaster prevention. In 2019, a total of 58 chemical accidents were disclosed, but none were recorded in the Daegu area [33]. Reporting and monitoring systems are mainly focused on industrial accidents such as overheating, damage to containers or valves, and transportation problems. If a gas-related event is suspected but the causative material or pollutant source is not specified, as in this case, management may not align with the current system. In the case of the hydrofluoric acid leak in Gumi, despite some confusion present at the time, actions after the accident were based on the chemical properties and toxicity of the hydrofluoric acid. This included the evacuation of nearby residents, a health impact assessment, and an environmental impact assessment [34]. In the present case, however, no measures were taken for asymptomatic persons, nearby residents, or anyone other than students with symptoms. The Ministry of Environment or other relevant government organizations must investigate, aggregate, and monitor suspected chemical accidents, even when the source and causative material are unclear.

The school is located in the Buk-gu region of Daegu. According to data from the 2019 Pollutant Release and Transfer Register, Buk-gu has 166,059 kg of annual air emissions from Korean group 1 chemicals (chemicals subject to emissions surveying with at least 1 ton consumed per year), while Daegu has a total of 1,284,542 kg [35]. Daegu’s Industrial Complex 3 is located near the school. Approximately 2,500 factories are located in Industrial Complex 3, and the metal processing product manufacturing industry accounts for approximately 50% of these. The industrial complex consists mainly of small enterprises, with approximately 80% of companies having 4 or fewer employees [36]. The 2019 emission levels of dimethylformamide, MEK, and toluene among VOCs in Industrial Complex 3 were 4,834; 6,695; and 5,322 kg, respectively [37]. Among air sampling data, MEK was detected relatively frequently at both industrial complexes and schools, which could indirectly show that schools can be affected by pollutants emitted from industrial areas. The concentrations of heavy metals (such as nickel and cadmium [38]) and VOCs (such as toluene, ethylbenzene, and xylene [39]) have also been reported to be higher in the vicinity of Industrial Complex 3 than in other regions of Daegu.

The first limitation of this investigation is that environmental medicine experts were excluded from the initial investigation and were only included 1 month later. Accordingly, police, not medical staff, performed the initial interview, and essential epidemiological information was not collected. Also, biological samples, such as urine and blood, were not collected and stored; thus, biomarker-based health effect and exposure assessments were not appropriately performed. Second, cooperation between ministries, such as those of environment, health, and education, was insufficient. Investigations and follow-up measures were primarily carried out by the environmental ministries. The educational institution’s actions in response to the students’ infringed rights to education were insufficient. No investigations or interventions were performed by health-related institutions other than the medical treatment of individuals, and the Ministry of Education paid only the treatment fees. Third, no discussion took place on the use of the Environmental Pollution Damage Relief Fund, an insurance fund established per the Act on Liability and Relief for Environmental Pollution Damage. This policy and fund were designed for the compensation of damage caused by environmental pollution. For pollution incidents, the identification of a specific cause or causal relationship may be difficult. However, the system is not appropriately applied, and the actual payout rate (or loss rate) from this fund is very low, at 3–6% [40]. Considering that the purpose of the Environmental Pollution Relief Act is to provide prompt compensation for damage in the event of an environmental pollution accident, the use of the relevant financial resources should be considered even if the cause is unknown. In this investigation, we argue that discussing environmental damage relief is necessary; however, due to a conflict of opinion among investigation team members, we conclude that the cause of the damage was unknown.

Although the investigation did not resolve differences of opinion about the cause, the investigation team did suggest measures to take after the accident. These suggestions included improving the air circulation system of the auditorium, improving the management of the science lab, conducting a health epidemiological survey among students, strengthening the management of nearby business sites, and strengthening the air pollution monitoring network in schools and residential areas. Implemented measures included hiring an additional public health teacher with a nursing license, installing air purifiers and air circulators in the auditorium, conducting a special inspection of pollutant-emitting factories at a nearby school, and installing a national monitoring network near the school. However, a longitudinal epidemiological survey, which is the most important measure to identify chronic health effects among students, was not performed.

In the present study, we conducted a descriptive investigation with temporal and spatial analysis. Although the source of the stench was not identified, suspected sources were narrowed down. Through this investigation, local government organizations and experts investigated cooperatively and established measures to prevent recurrence. As follow-up measures, the school environment can be improved by installing an air circulation system, inspecting pollutants at nearby factories, and installing additional measuring stations. However, fundamental measures to prevent recurrence in consideration of students’ rights to health and education have not been discussed, and public discussion in society and academia has not been sufficiently developed. Although more than 100 patients were affected, the incident was not reported as a chemical accident investigated by the Ministry of Environment, since the source was unclear. Only some local media reported the incident, and limited literature and media coverage exists to provide information for similar incidents in the future. Therefore, the current study is expected to become an academic reference based on its examination of the strengths and limitations of the investigation.

Here, we share our investigation process. We endeavored to suggest the most likely causes of the stench, including toxic puff gas generated by a nearby industrial complex. However, such incidents have not been sufficiently discussed in the literature or among the public. Therefore, herein we review the investigation process, focusing on its strengths and limitations. We expect these data to ensure that more appropriate measures can be taken in similar cases in the future.

## Figures and Tables

**Figure f5-epih-44-e2022105:**
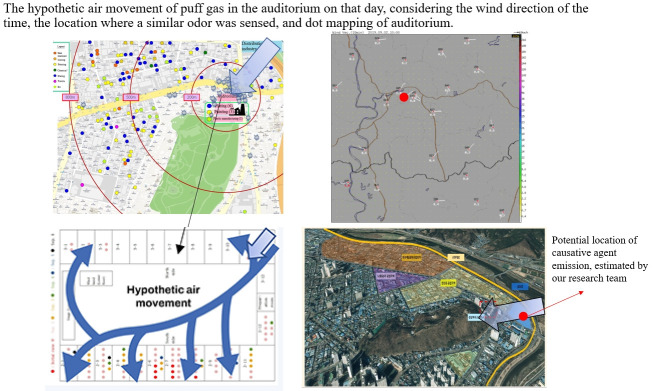


**Table 1:** Hourly timetables of the incident day

Time	Activity	Notes
September 2nd		
7:50–8:10	Auditorium cleaning (upper floor) Wind band practice (lower floor)	Windows opened No symptoms or odor
8:10–9:00	Physical education class (lower floor)	No symptoms or odor
9:20–9:40	Students enter the auditorium Seven patients develop symptoms	Stench was noticed on upper floor
9:40–10:00	Teachers enter the auditorium Entrance ceremony is held	Few people sensed stench
10:05–10:50	Students return to class Symptoms persisted among initial 7 patients, who visited emergency room via ambulance	
11:00–13:00	Arrival of National Emergency Management Agency, police, scientific investigation team, Gas Safety Corporation, media, etc. All students were screened for symptoms, and a total of 74 patients were identified	Inspection of school facilities (including science laboratory) No specific problems identified
13:00–14:00	Meeting, briefing, and air sampling	
14:50	Students go home	

**Table 2:** Similar cases identified in interviews and records

Time	Event	Action
Sep 22, 2017	Stench occurs (18:00)	Students in dormitory and classrooms returned home^1^ Potential causes investigated in nearby areas
Sep 28, 2017	Stench occurs (18:00)	Students in dormitory and classrooms returned home
Oct 11, 2017	Stench occurs (11:00)	-
Oct 30, 2017	Stench occurs (18:00)	Students in dormitory and classrooms returned home
Oct 31, 2017	Stench occurs (17:40)	-
Nov 2, 2017	Stench occurs (17:30)	Potential causes investigated in nearby areas Investigation of nearby factories planned
Apr 17, 2018	Stench occurs (18:35)	Potential causes investigated in nearby areas
Apr 17, 2018	Stench occurs (18:35)	-
Sep 7, 2018	Stench occurs	Potential causes investigated in nearby areas

1 Korean high school students usually remain at school and study until 21:00.

**Table 3:** Stench patterns summarized from the interviews of 74 students (including duplicates)

Stench pattern	n (%)
Burning odor	48 (64.9)
Metallic	8 (10.8)
Rubber	5 (6.8)
Plastic	10 (13.5)
Non-specific	25 (33.8)
Gas	18 (24.3)
Chemicals	12 (16.2)
Paint or solvent	7 (9.5)
Other^1^	16 (21.6)

1 Other: irritant, adhesive, bitter, soap bubble, vinyl, bleach (chlorax), smoky, unpleasant, wax, oil, rotten; Since the questionnaire was unstructured, many descriptions were unclear, and each participant could answer with more than 1 response.
