# Supplementary material for: An outbreak of neurologic symptoms among patients exposed to an unknown stench in a high school near an industrial complex: an epidemiological investigation
Source: Epidemiol Health. 2022 Nov 9;44:e2022105. doi: 10.4178/epih.e2022105 (PMC10111089; doi:10.4178/epih.e2022105)
Supplement: Supplementary file 1 [file epih-44-e2022105-Supplementary-1.docx]

**1. Authority, location, time and method of air sampling and measurement,**

(1) Institute of Health and Environment Research, Daegu

Sep 6: Measurement of ultrafine dust, carbon dioxide, carbon monoxide, total airborne bacteria, formaldehyde, and total volatile organic compounds (14:40)

September 6, 8, 15: Sample collection analysis inside and outside the auditorium (Sep 6 15:00~17:00, Sep 8 17:30~19:00, Sep 15 17:30~19:00) 3 points inside the auditorium and 1 point outside the auditorium. Analysis for 105 volatile organic substances

September 9: On-site inspection of mobile vehicles in nearby general industrial areas and the high school

(2) Korea Environment Corporation

Sep 5-6: A total of 73 types (designated odorous substances + volatile organic compounds) were measured with the Hazardous Air Pollutants (HAPs) mobile measurement system (SIFT-MS). mobile measurement (Sep 5, 14:00 - 16:25), mobile movement measurement (Sep 6 10:17 - 12:42), fixed point continuous measurement (Sep 5 10:03 – Sep 6 10:05).

Sep 19 – Sep 20: Continuous measurement of 16 types of volatile organic compounds using a manual HAPs sampling device (STS25) for hazardous air substances (school field, auditorium, science lab) 24 hours a day Science lab/auditorium (Sep 19 12:00 – Sep 20 12:00, School field Sep 19 13:00-Sep 20 13:00)

(3) National Institute of Environmental Research

Sep 23 – 25: A total of 7 points including the science lab, auditorium, were sampled. Airs from science lab and auditorium were sampled with bag. The surrounding air quality is analyzed on-site with a mobile measurement system using mass spectrometry.

(4) Daegu National Forensic Service

Sep 2: Gas collection in science lab, in front of the auditorium, on the 2nd floor of the auditorium with colorless Tedler bag. Gas collection in two reagent cabinets and the exhaust fan outside the reagent room with silver Tedler bag. Analysis was performed by GC-MS, TCD.

**2. Results of air measurement by authority**

1. Result from Institute of Health and Environment Research, Daegu

(1) Sep 6

*Unit: ppb

| Parameter | Units | Lower floor of auditorium | Upper floor of auditorium | Science lab |
| --- | --- | --- | --- | --- |
| PM10 | ug/m3 | 22 | 21.9 | 18.7 |
| PM2.5 | ug/m3 | 16.8 | 13 | 15.9 |
| CO2 | ppm | 718 | 506 | 544 |
| CO2 | ppm | 1.3 | 1.7 | 3.2 |
| Airborne bacteria | CFU/m3 | 345 | 397 | 89 |
| formaldehyde | ug/m3 | 22.2 | 18.3 | 121.2 |
| VOC | ug/m3 | 199.9 | 142.6 | 570.1 |

(2) Sep 6-15

*Unit: ppb

|  | Outside of the auditorium(1) | Outside of the auditorium(2) | Lower floor of auditorium | Upper floor of auditorium | Science lab |
| --- | --- | --- | --- | --- | --- |
| 1,3-butadiene | 20.3 | 12.1 | 39.9 | 90.6 | 91.6 |
|  |  |  | 60.8 | 66.5 | 89.9 |
|  | 7.3 |  | 25.4 | 25.9 | 70.4 |
| Acetaldehyde | 237 | 161 | **250** | **271** | **302** |
|  |  |  | 32 | 34 | 0 |
|  | 28 |  | 41.3 | 70.3 | 73.3 |
| Acetonitrile | 24.2 | 17.5 | 43.6 | **884** | **323** |
|  |  |  | 4.6 | 6 | 16.4 |
|  | 5.9 |  | 0 | 0 | 0 |
| Carbonyl sulfide | 18.9 | 13.9 | 20.1 | **414.5** | 160.5 |
|  |  |  | 0 | 0 | 11.8 |
|  | 0 |  | 0 | 0 | 0 |
| Ethanol | 43.5 | 29.7 | 104.8 | 69.8 | **1105.8** |
|  |  |  | 10.7 | 0 | **563** |
|  | 0 |  | 0 | 10.9 | 107.8 |
| Ethyl chloride | 12.7 | 0 | 31.8 | 21.4 | 366 |
|  |  |  | 0 | 0 | 174.6 |
|  | 10.3 |  | 6.7 | 10.6 | 44.4 |
| Isobutane | 130.9 | 88.9 | 88.9 | 98.9 | 182.9 |
|  | 0 |  | 0 | 379 | 341.9 |
|  | 0 |  | 0 | 0 | 33.8 |
| Isopentane | 22.3 | 16.3 | 20.7 | 168.5 | 108.5 |
|  |  |  | -10.8 | 11.4 | 146.8 |
|  | 0 |  | 0 | 0 | 15.1 |
| Methanol | 17.2 | 10.9 | 33.6 | 164.1 | **293.1** |
|  |  |  | 54.6 | 59.9 | **470.5** |
|  | 5.6 |  | 20.7 | 21.7 | 59.3 |
|  |  |  |  |  |  |

Sep 9 – Sep 10

*Unit: ppb

|  |  | Industrial complex area |  |
| --- | --- | --- | --- |
|  | School | zone 1 | zone 2 |
| Isobutane | 16.4 (1.3-336) | 20.4 (4.4-330) | (-) |
| Methanol | 11.8 (2.5-120) | 29.6 (8.5-270) | 21.1 (10-135) |
| Propanoic acid | (-) | 6.6 (0-37.1) | (-) |
| Xylene + Ethylbenzene | (-) | 4.3 (0-30.4) | (-) |
| Acrylic acid | (-) | (-) | 24.8 (2.9-183) |
| Butanoic acid | (-) | (-) | 8.1 (1.5-20.7) |
| Butanone | (-) | (-) | 18.5 (1.4-221) |
| Toluene | (-) | (-) | 27.7 (2.8-291) |
| 1-butene | 3.5 (3.5-59.2) | (-) | (-) |
| 2-propanol | 1.9 (1.9-76.6) | (-) | (-) |
| Acetone | 7.3 (7.3-63.1) | (-) | (-) |
| Acrolein | 4.2 (4.2-156) | (-) | (-) |
| Ethanol | 18.9 (18.9-123) | (-) | (-) |

Zone 1: About 140 including plating industry, painting drying, eyeglass industry, etc.

Zone 2: Including Industrial Complex 3, including machinery, electricity and electronics, and transportation

1. Result from Korea Environment Corporation

Sep 5-6

*Unit: ppb

| Parameter | 1st mobile measurement  (Sep 5) | 2nd mobile measurement  (Sep 6) | Fixed  (Sep 5-6) |
| --- | --- | --- | --- |
| Ammonia | 0.13 | 0.2 | 0.06 |
| Trimethylamine | 0.36 | 0.24 | 0.16 |
| hydrogen sulfide | 0.39 | 0.35 | 0.37 |
| methyl mercaptan | 0.09 | 0.05 | 0.06 |
| dimethyl sulfide | 0 | 0 | 0 |
| dimethyl disulfide | 0.05 | 0.02 | 0.03 |
| acetaldehyde | 0.51 | 1 | 0.64 |
| propionaldehyde | 0.35 | 0.05 | 0.1 |
| butylaldehyde | 0.18 | 0.17 | 0.17 |
| n, iso-valeraldehyde | 0.26 | 0.22 | 0.23 |
| styrene | 0.01 | 0.01 | 0 |
| toluene | 0 | 0 | 0 |
| o.m.p-xylene | 0 | 0 | 0 |
| MEK | 0.35 | 0.23 | 0.25 |
| MIBK | 0.29 | 0.17 | 0.19 |
| butyl acetate | 0.22 | 0.12 | 0.1 |
| iso-butyl alcohol | 1.09 | 0.9 | 0.8 |
| propanoid acid | 0.15 | 0.04 | 0.04 |
| butyric acid | 0.17 | 0.07 | 0.09 |
| n.isovaleric acid | 1.26 | 0.7 | 0.9 |
| 1.3-dichloropropene | 0.13 | 0.1 | 0.05 |
| 1.2.4-trichlorobenzene | 0 | 0 | 0 |
| 1.2-dibromoethane | 0.03 | 0.01 | 0 |
| 1.2-dichloropropane | 1.1 | 0.15 | 0.19 |
| 1.3-butadiene | 0.04 | 0.02 | 0.01 |
| 1-butene; cis-2-butene; trans-2-butene | 3.13 | 0.91 | 1.91 |
| 1-hexene | 0.17 | 0.03 | 0.02 |
| 1-pentene; cis-2-pentene; trans-2-pentene | 0.44 | 0.14 | 0.27 |
| 2-methylheptane; 3-methylheptane; 2,2,4-trimethylpen | 0.29 | 0.35 | 0.42 |
| 2-methylhexane;3-methylhexane;2,3-dimethylpentane | 0.36 | 0.45 | 0.45 |
| Freon 11 | 1.16 | 0.31 | 0.34 |
| Freon 114; Freon 113 | 0.07 | 0.03 | 0.01 |
| Freon 12;Freon 113 | 1.93 | 0.81 | 0.8 |
| Acetylene | 0.07 | 0.04 | 0.04 |
| Acrylonitrile | 0.22 | 0.05 | 0.05 |
| Benzene | 0.07 | 0.04 | 0.07 |
| Benzyl chloride | 0.07 | 0.03 | 0.04 |
| Carbon disulfide | 1.35 | 0.02 | 0.04 |
| Carbon tetrachloride | 0.02 | 0.01 | 0.01 |
| chlorobenzene | 0.51 | 0.19 | 0.15 |
| chloroform; dichloromethane; tetrachloroethane | 0.36 | 0.04 | 0.03 |
| cyclohexane | 0.01 | 0.01 | 0.01 |
| cyclopentane | 0.04 | 0.01 | 0.01 |
| dichlorobenzene | 0.12 | 0.03 | 0.05 |
| dichloroethylene (1.1- and 1.2-) | 0.29 | 0.08 | 0.11 |
| diethylbenzene | 0.08 | 0.02 | 0.02 |
| ethene | 0 | 0 | 0 |
| ethyl chloride; 1.1-dichloroethane | 0.03 | 0.01 | 0.01 |
| hexachlorobutadiene | 0.02 | 0.01 | 0.01 |
| isobutane | 0.36 | 0.2 | 0.31 |
| isohexane | 0.5 | 0.49 | 0.58 |
| isopentane | 0.61 | 0.72 | 0.64 |
| isoprene | 0.08 | 0.02 | 0.04 |
| m.o.p-ethyltoluene;isopropylbenzene;propylbenzene; | 0.49 | 0.35 | 0.53 |
| methyl bromide | 1.32 | 0.91 | 1.12 |
| methyl chloride | 0.14 | 0.05 | 0.1 |
| methyl cyclohexane | 2 | 0.37 | 0.67 |
| methylcyclopentane | 0.01 | 0 | 0.01 |
| n-butane | 1.93 | 0.22 | 0.19 |
| n-decane | 0.73 | 0.29 | 0.43 |
| n-dodecane | 0.38 | 0.26 | 0.42 |
| n-heptane | 0.49 | 0.22 | 0.19 |
| n-hexane | 0.39 | 0.19 | 0.16 |
| n-nonane | 0.13 | 0.04 | 0.06 |
| n-octane | 0.39 | 0.32 | 0.26 |
| n-pentane | 2.86 | 1.46 | 1.35 |
| n-undecane | 0.59 | 0.39 | 0.37 |
| propane | 1.48 | 0.01 | 0.03 |
| propene | 0 | 0 | 0 |
| tetrachloroethylene | 0.01 | 0 | 0 |
| trichloroethane (1.1.1- and 1.1.2-) | 0.02 | 0.01 | 0 |
| trichloroethylene | 0.09 | 0 | 0.02 |
| vinyl chloride; 1.2-dichloroethane | 0.12 | 0.01 | 0.01 |

Sep 19-22

*Unit: ppb

| Parameter | Auditorium | Science lab | School field | Average result of Daegu,Jan to July |
| --- | --- | --- | --- | --- |
| Benzene | 0.082 | 3.009 | ND | 0.375 |
| Toluene | 5.253 | 5.968 | 1.6 | 5.807 |
| Ethyl benzene | 1.04 | 0.957 | 0.413 | 0.447 |
| m,p-Xylene | 1.703 | 2.109 | 0.62 | 0.724 |
| Styrene | 0.143 | 0.323 | 0.051 | 0.036 |
| o-Xylene | 0.492 | 0.736 | 0.159 | 0.231 |
| Chloroform | 0.043 | 0.045 | 0.023 | 0.025 |
| Methyl chloroform | ND | ND | ND | 0.001 |
| Trichloroethylene | 0.081 | 0.036 | 0.035 | 0.134 |
| Tetrachloroethylene | 0.004 | ND | ND | 0.016 |
| 1,1-Dichloroethane | ND | ND | ND | 0 |
| Carbon tetrachloride | 0.113 | 0.17 | 0.044 | 0.085 |
| 1,3-Butadiene | ND | 0.732 | 0.001 | 0.041 |
| Methylene chloride | 0.423 | 13.704 | 0.091 | 0.82 |
| Vinyl Chloride | ND | ND | ND | 0.039 |
| 1,2-Dichloroethane | 0.012 | 0.041 | ND | 0.039 |

(3) Result from National Institute of Environmental Research

*Unit: ppb

|  | Science lab | Auditorium | Mobile measurement  (1) | Mobile measurement  (2) | Mobile measurement  (3) | Mobile measurement  (4) |
| --- | --- | --- | --- | --- | --- | --- |
| Benzene | 0.24 | 0.32 | 0.55 | 0.7 | 3.3 | 1.32 |
| Toluene | 15.92 | 41.11 | 133.25 | 252.61 | 292.92 | 499.68 |
| Xylene | 21.28 | 85.41 | 102 | 175.48 | 829.74 | 205.14 |
| Ammonia | 2.62 | 21.26 | 45.61 | 70.26 | 57.98 | 20.44 |
| Acetaldehyde | 10.18 | 18.51 | 12.32 | 14.41 | 11.67 | 10.15 |
| MEK | 2.1 | 14.84 | 29.92 | 41.43 | 16.83 | 44.61 |
| Stylene | 3.01 | 6.76 | 5.71 | 11.01 | 57.69 | 4.44 |

1. Result from Daegu National Forensic Service

| Location | Result |
| --- | --- |
| Science lab | Toluene (+), Xylene (+) |
| Chemical cabinet | Toluene (+), Xylene (+) |
| In front of the auditorium entrance, on the 2nd floor of the auditorium, inside the reagent room on the left, outside the reagent room exhaust fan | no VOC |
