# Supplementary material for: An outbreak of neurologic symptoms among patients exposed to an unknown stench in a high school near an industrial complex: an epidemiological investigation
Source: Epidemiol Health. 2022 Nov 9;44:e2022105. doi: 10.4178/epih.e2022105 (PMC10111089; doi:10.4178/epih.e2022105)
Supplement: Supplementary file 2 [file epih-44-e2022105-Supplementary-2.docx]

| Supplementary Material 1. Daily timetable of the days after the incident occurred | | | | | | |
| --- | --- | --- | --- | --- | --- | --- |
|  | Event | Action | Authority institution | Patient incidence (without duplicates)* | Left school early | Absent |
| September 2 | Incident occurred | Air sampling | Institute of Health and Environment, Daegu National Forensic Service | 74 | 0 | 0 |
|  |  | School facility investigation | Police |  |  |  |
| September 3 |  | Interview of 74 patients from September 2 | Police | 17 | 1 | 2 |
| September 4 |  |  |  | 9 | 8 | 5 |
| September 5 |  | Air sampling via portable (bus) air measurement device in industrial complex near school | National Institute of Environmental Research | 4 | 5 | 2 |
| September 6 |  | Air sampling in auditorium | Institute of Health and Environment Research, Daegu | 1 | 3 | 3 |
|  |  | Air sampling via portable (bus) air measurement device in industrial complex near school | National Institute of Environmental Research |  |  |  |
|  |  | Proposal of a joint investigation team at a working-level meeting due to the failure of the relevant agency to identify the cause | National Forensic Service, Fire Department, Gas Safety Corporation, police, Meteorological Administration, Environment Agency, Daegu municipality, etc. |  |  |  |
| September 7 |  |  |  |  | - | - |
| September 8 |  | Air sampling in auditorium | Institute of Health and Environment Research, Daegu | - | - | - |
| September 9 |  | Monitoring with measuring vehicle | Institute of Health and Environment Research, Daegu | - | - | - |
| September 10 |  | - | - | - | - | - |
| September 11 |  | - | - | - | - | - |
| September 15 |  | Air sampling in auditorium | Institute of Health and Environment Research, Daegu | - | - | - |
| September 16 | Burning odor (library) | Special inspection of air emission factories (through approximately September 27) | Regional Environment Agency, Office of Education, Daegu municipality | - | - | - |
| September 18 | Burning odor (entrance) |  |  | - | - | - |
| September 19 |  | Air sampling in science laboratory and playground | Korea Environment Corporation | - | - | - |
| September 20 | Burning odor  (information room, on the way to school) | Air sampling in science laboratory and playground | Korea Environment Corporation | - | - | - |
| September 23 |  | Monitoring with measuring vehicle | National Institute of Environmental Research | - | - | - |
| September 24 | Burning odor (3-11 class) | Monitoring with measuring vehicle | National Institute of Environmental Research | - | - | - |
| September 25 |  | Air sampling via portable (bus) air measurement device in industrial complex near school | National Institute of Environmental Research | - | - | - |
| September 26 |  | Air sampling via portable (bus) air measurement device in industrial complex near school | National Institute of Environmental Research | - | - | - |
| September 27 |  | Joint investigation team launched | Investigation team (7 members including 1 medical staff member) |  |  |  |
| September 30 | Burning odor (3-12 class) |  |  | - | - | - |
| October 2 | Field investigation | Additional interview, field investigation | Environmental medical staff (our research team) | - | - | - |
| October 3 | Burning odor (principal's office, playground) |  |  | - | - | - |
| October 16 |  | Joint investigation team meeting  Debate on internal and external factors  Suggestion of the need for review of medical records, etc. | Investigation team^†^ (7 members including 1 medical staff member) | - | - | - |
| October 17 |  | Review of medical records and interview records (through approximately November) | Environmental medical staff (our research team) | - | - | - |
| November 13 |  | Joint investigation team meeting  Disagreement among investigators | Investigation team (7 members including 1 medical staff member) | - | - | - |
| January 10, 2020 |  | Announcement of survey results Disagreement among investigators continues; case closed with cause unknown | Investigation team (7 members including 1 medical staff member) | - | - | - |
|  |  |  |  |  |  |  |
| *Includes visits to clinics or hospital via outpatient care, hospitalization, or emergency room due to neurologic symptoms. On September 7-11, no cases were reported. Since then, no investigation has occurred. | | | | | | |
| †The research team consisted of 7 members: 2 air environment experts, 1 chemical expert, 1 industrial hygiene expert, 1 sampling and analysis expert, 1 medical expert, and 1 non-government organization member. | | | | | | |
