# Supplementary figures and images for: An outbreak of neurologic symptoms among patients exposed to an unknown stench in a high school near an industrial complex: an epidemiological investigation
Source: Epidemiol Health. 2022 Nov 9;44:e2022105. doi: 10.4178/epih.e2022105 (PMC10111089; doi:10.4178/epih.e2022105)

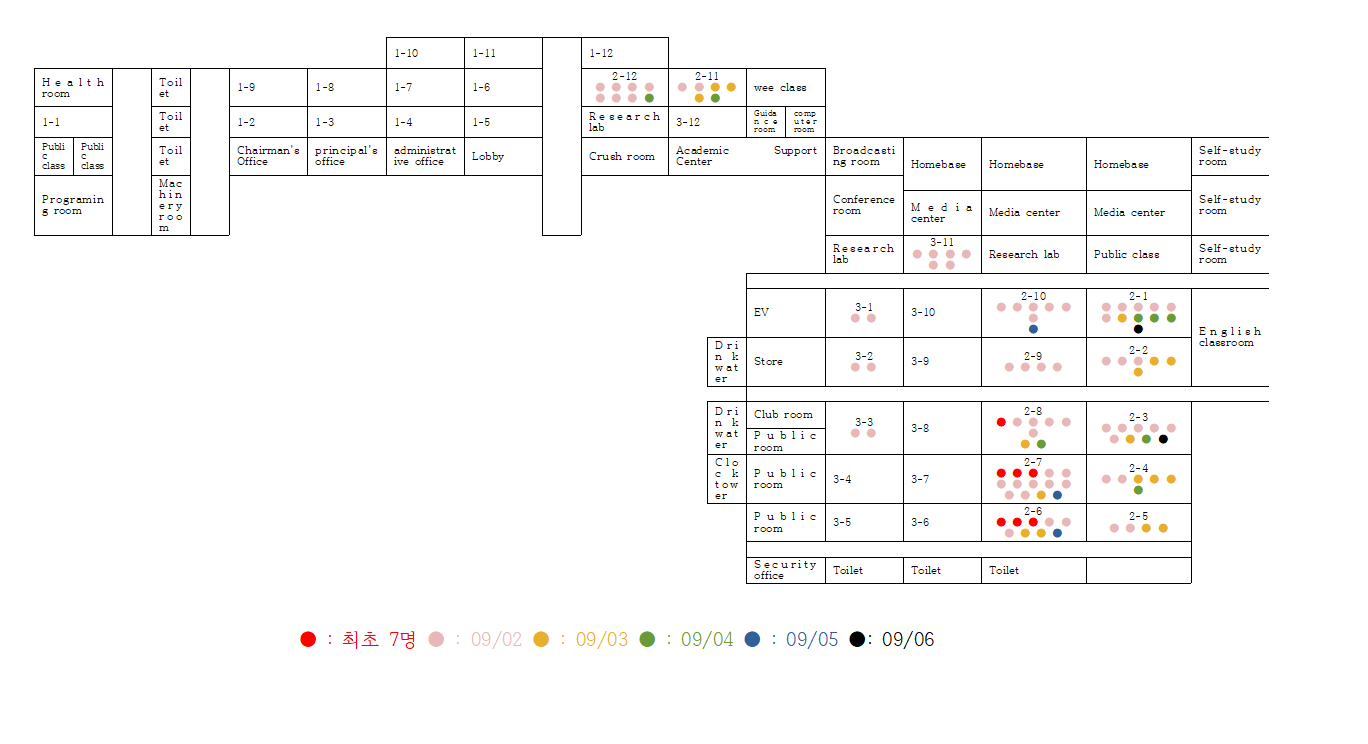

Supplement: Supplementary file 3 [file epih-44-e2022105-Supplementary-3.tif]
