# Supplementary material for: An outbreak of neurologic symptoms among patients exposed to an unknown stench in a high school near an industrial complex: an epidemiological investigation
Source: Epidemiol Health. 2022 Nov 9;44:e2022105. doi: 10.4178/epih.e2022105 (PMC10111089; doi:10.4178/epih.e2022105)
Supplement: Supplementary file 5 [file epih-44-e2022105-Supplementary-5.pptx]

## Slide 1
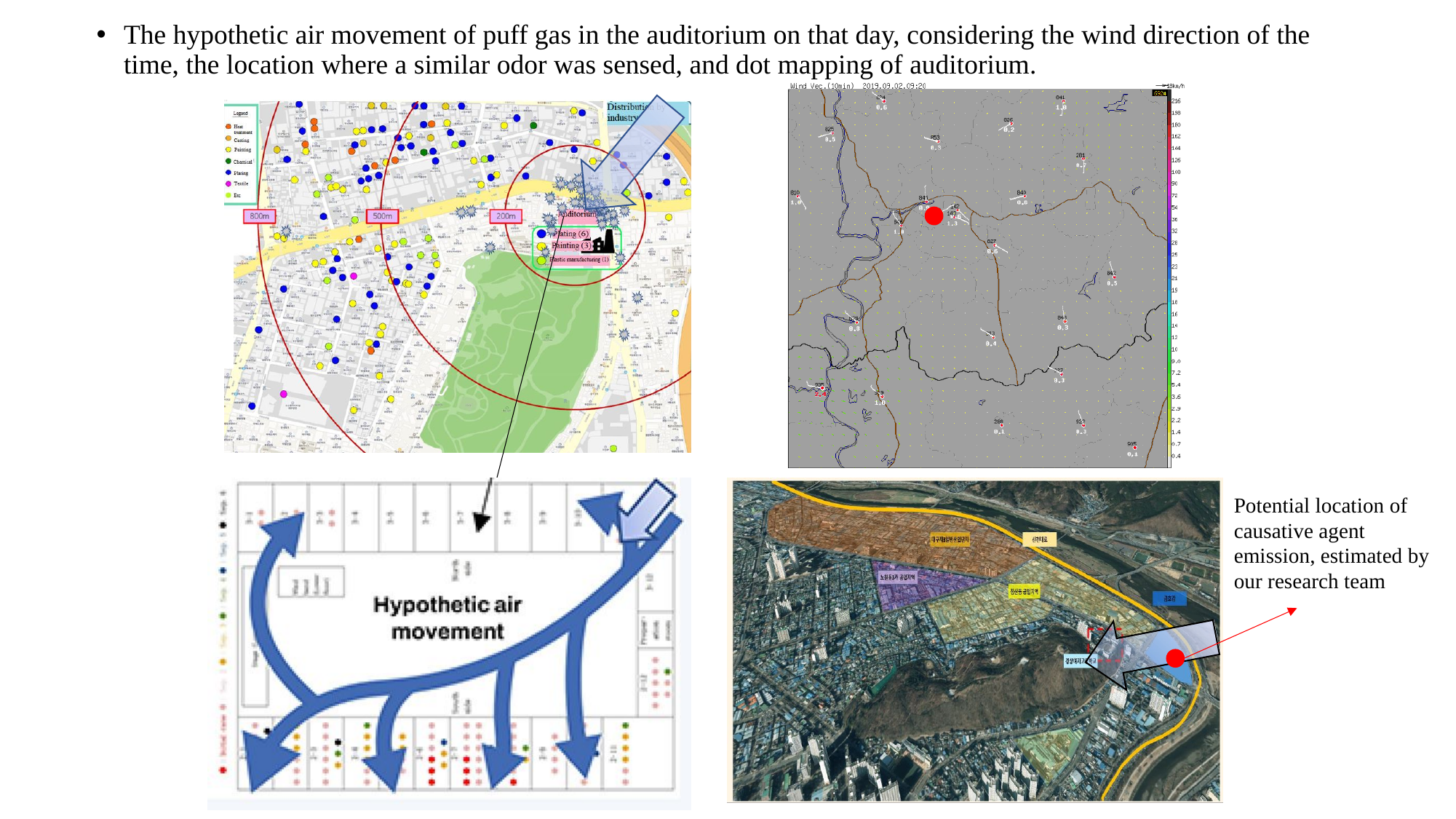

The hypothetic air movement of puff gas in the auditorium on that day, considering the wind direction of the time, the location where a similar odor was sensed, and dot mapping of auditorium.
Potential location of
causative agent
emission, estimated by
our research team
